# Supplementary material for: Comparison of mortality and hospitalizations of older adults living in residential care facilities versus nursing homes or the community. A systematic review
Source: PLoS One. 2023 May 31;18(5):e0286527. doi: 10.1371/journal.pone.0286527 (PMC10231833; doi:10.1371/journal.pone.0286527)
Supplement: S1 Appendix — (DOCX) [file pone.0286527.s002.docx]

Appendix 1

Search strategy to find potential relevant articles for inclusion in the review of mortality and hospitalizations of older adults living in residential care facilities versus nursing homes or communities

| **Pubmed** |
| --- |
| (("senior housing"[Title/Abstract]) OR ("independent living communities"[Title/Abstract]) OR ("assisted living facilities"[Title/Abstract]) OR ("continual care retirement communities"[Title/Abstract]) OR ("residential care"[Title/Abstract]) OR ("residential cares"[Title/Abstract]) ) AND (("hospitalization"[All fields]) OR ("hospitalizations"[All fields]) OR ("death"[All fields]) OR ("mortality"[All fields])) |
| **Scopus** |
| **Web of Science** |
