# Supplementary material for: Comparison of mortality and hospitalizations of older adults living in residential care facilities versus nursing homes or the community. A systematic review
Source: PLoS One. 2023 May 31;18(5):e0286527. doi: 10.1371/journal.pone.0286527 (PMC10231833; doi:10.1371/journal.pone.0286527)
Supplement: Attachment — Submitted filename: Response to editor comments_15.05.23.docx [file pone.0286527.s004.docx]

**Comments to the editor:**

As the editor assigned to your submission, I have carefully reviewed your responses to the reviewers' comments. While I believe your responses are appropriate, I would like to provide you with some additional feedback for further clarification. Please find my comments below:

1. *There seems to be some confusion in the article regarding patients living in the community. The abstract and inclusion criteria are not clear in this regard. I only understood what you did in relation to this patient group when reading the later part of the results section.*

**Authors comment:** We would like to thank the editor for raising these points in order to improve the quality of the article . We have compared the mortality and hospitalizations rates of older people living in residential care facilities or in the community. We agree with you and we have clarified this point in the abstract and inclusion criteria, using only the term “communities” throughout the article.

*2. According to the PRISMA and AMSTAR2 criteria, authors are required to provide an example of their search strategy for at least one of the investigated bibliographic databases.*

**Authors comment:** We have added an example of our strategy based on Pubmed in an additional file.

*3. Any limitations in the search process should be justified, including limitations on the language of articles. There are scientific references available that can be cited to support this argument (e.g., doi: 10.1017/S0266462312000086).*

**Authors comment:** We have added in the limitation section “It has been established that the exclusion of non-English language articles has only a minimal effect on the overall conclusions of the reviews” with the following recent reference:

Nussbaumer-Streit B, Klerings I, Dobrescu A, et al. Excluding non-English publications from evidence-syntheses did not change conclusions: a meta-epidemiological study. J Clin Epidemiol 2020;118:42–54.

*4. Data extraction is typically expected to be performed in duplicate. Please acknowledge this as a limitation of the study.*

**Authors comment:** We have added this limitation as suggested.

*5. Has the assessment of study quality been conducted in duplicate?*

**Authors comment:** the quality assessment of the study was conducted by a senior methodologist and not in duplicate. We have added this as a limitation.

*6. The "Statistical Analysis" paragraph appears to be incomplete. It is important to explain why a meta-analysis was not conducted.*

**Authors comment:** We agree with this comment and explain why we did not perform a meta-analysis in the statistical analysis:

“After reviewing each of the studies included in this review, we considered that a meta-analysis was not relevant, due to the low methodological quality of the studies, the diversity of study designs and follow-up and the heterogeneity of populations, for which essential baseline characteristics were not available.”

*7. I would like the research question to be formulated using the PICO format.*

**Authors comment:** We agree with you and formulated the research question using the PICO format in the method section (eligibility criteria) as proposed by the PRISMA guidelines:

“Population: We only selected cohort studies conducted among older persons (≥65 years old).

Intervention: We selected studies that had a cohort design (prospective or retrospective cohort design) and at least six months of follow-up and that measured mortality or hospitalizations as outcomes. For duplicate publications from the same cohort, we selected those with the largest number of participants. We excluded cross-sectional studies and, reviews.

Comparison: We selected cohort studies comparing residential care facilities with nursing homes and/or communities,

Outcomes: To be included, we considered studies that reported the number of participants or person years and the number of deaths or hospitalizations in both groups (residential care facilities versus nursing home and/or communities). We excluded studies reporting mortality or a hospitalization for a specific medical condition.”

We hope that these changes meet your expectations.
